# Evaluation of air polishing with a sterile powder and mechanical debridement during regenerative surgical periimplantitis treatment: a study in dogs

**DOI:** 10.1007/s00784-020-03572-2

**Published:** 2020-09-10

**Authors:** Alex Solderer, Benjamin E. Pippenger, Marcel Donnet, Daniel Wiedemeier, Liza L. Ramenzoni, Patrick R. Schmidlin

**Affiliations:** 1 Clinic of Conservative and Preventive Dentistry, Division of Periodontology and Peri-implant Diseases, Center of Dental Medicine, University of Zurich, Plattenstrasse 11, 8032 Zurich, Switzerland; 2 Preclinical & Translational Research Group, Straumann AG, Basel, Switzerland; 3 EMS, Nyon, Switzerland; 4 Statistical Services, Center of Dental Medicine, University of Zurich, Zurich, Switzerland

**Keywords:** Dental implants, Periimplantitis, Guided bone regeneration, Air polishing, Dehiscence defect

## Abstract

**Objectives:**

To evaluate the effectiveness of mechanical debridement and/or air polishing on the healing of ligature-induced buccal periimplantitis dehiscence defects in dogs.

**Material and methods:**

Forty-eight implants were placed in the mandibles of twelve beagle dogs, and periimplantitis was induced for 2 months using ligatures. The resulting buccal dehiscence-type defects were surgically cleaned and augmented (xenogenic filler and resorbable membrane) according to one of the following treatments: (1) Cleaning with carbon curette (debridement - D) and guided bone regeneration (GBR/G): DG, (2) air polishing cleaning (A) and GBR: AG, (3) a combination of D/A/G: DAG, and (4) D/A without GBR: DA. After 2 months, histomorphometric and inflammatory evaluations were conducted.

**Results:**

The median bone gain after therapy ranged between 1.2 mm (DG) and 2.7 mm (AG). Relative bone gain was between 39% (DG) and 59% (AG). The lowest inflammation scores were obtained in DA without GBR (5.84), whereas significantly higher values between 8.2 and 9.4 were found in the groups with augmentation. At lingual sites without defects, scores ranged from 4.1 to 5.9. According to ISO, differences above 2.9 were considered representative for irritative properties.

**Conclusions:**

All treatments resulted in partial regeneration of the defects. No treatment group showed a significantly (*p* < 0.05) better outcome. However, pretreatment with air polishing showed a tendency for less inflammation. Noteworthy, inflammation assessment showed an overall irritative potential after GBR in the evaluated early healing phase.

**Clinical relevance:**

Periimplantitis treatment still represents a big issue in daily practice and requires additional preclinical research in order to improve treatment concepts.

## Introduction

The use of implants in dental practice has become a routine procedure in order to replace one or more missing teeth using either fixed or removable dentures. High success rates of up to 97% after 10 [1] and 75% over 20 years [2] underline the excellent applicability of this treatment. However, prosthetic and biological complications may cast a cloud over this enthusiastic impression. Especially periimplant inflammations diagnosed as mucositis and periimplantitis should be mentioned in the first place [3]. They are specified as plaque-associated pathological conditions affecting the soft and hard tissues around dental implants, resulting in inflammation of the mucosa and progressive periimplant bone loss [4]. Periimplantitis represents one of the main reasons for late implant failure [5, 6] and a metaanalysis estimated a weighted mean prevalence of 22% (CI: 14–30%) for disease development [7]. Like periodontitis, periimplantitis is mainly caused by pathogen biofilms but may also be modulated by different cofactors [8].

Different approaches can be found for the treatment of implants with periimplantitis, and a plethora of surgical methods, techniques, and materials applied during such interventions have been described in the literature so far [9]. Regardless the existence of many different techniques, two treatment parameters remain common to periimplant treatment procedures: (i) cleaning and decontamination of the affected implant surfaces, i.e., calculus, biofilm, and endotoxin (4) and—in the case of regenerative procedures—(ii) the stable augmentation and coverage of the defects, which includes different possible filler materials and membranes with and without additional biologic agents [10]. Not surprisingly, many different combinations with regard to techniques and materials used are reported in the literature [9], making a comparison of the outcomes, an elaboration of appropriate clinical treatment recommendations and/or defining reliable standards very difficult.

With regard to the cleaning of a contaminated surface, several physico-chemical antiinfective methods can be used including conventional mechanical debridement with hand and/or (ultra)sonic instruments lasers and photodynamic therapy [9]. In addition, air polishing represents an alternative or adjunct option [9, 11, 12]. A recent consensus statement claimed that air polishing represents a valuable alternative for effectively disrupting bacterial biofilms [11]. Among the materials applied for this approach, glycine and sodium bicarbonate powders were found to be equally effective in biofilm removal [11]. Clinically, air polishing was shown to result in significantly higher reduction of bleeding-on-probing (BOP) [11], even in a nonsurgical periimplantitis treatment protocol as compared with mechanical debridement or ER:YAG laser monotherapies [11]. However, without surgical access, none of the non-surgical concepts may achieve complete and predictable disease resolution so far [11]. Therefore, adequate decontamination of the infected implant surfaces to a critical level remains essential, especially when regenerative concepts are applied in order to allow for successful graft integration [9].

Regarding periimplantitis-derived bone defect regeneration, the use of air polishing in combination with guided bone regeneration (GBR) procedures has been already described in clinical studies and a recent review, in which air polishing techniques showed beneficial effects [12]. Since conclusive statements on the preclinical and/or clinical applicability of air polishing for the management of periimplantitis diseases remains elusive, additional studies are warranted to clarify the additional benefits.

The present animal study aimed to assess the healing after different cleaning options including debridement with carbon curettes and/or air polishing when treating periimplantitis buccal dehiscence defects with or without simultaneous GBR. According to the author’s knowledge, this is the first preclinical study using a sterilized air polishing powder (sodium bicarbonate with a grain size ranging from 10–25 microns) applied with a specialized delivery system unit (prototype designed for sterile usage). Histomorphometric evaluations were conducted in order to measure bone gain and the inflammatory infiltrate (ISO 10993) [13]. It was hypothesized that air polishing leads to more bone formation and less inflammation due to improved cleaning of the affected implant surfaces.

## Material and methods

### Animals and anesthetic protocol

In the present animal study, twelve adult female Beagle dogs were included. They were all systemically healthy, 12–13 months old and with an average weight of 10 kg at the day of surgery. During the entire study, dogs were fed with soft food and water.

The study was conducted at the BWEF, NJ, USA. The animals were housed in individual cages under controlled environment. This study was performed in accordance with applicable US laws regarding the treatment of experimental animals and under the ethical approval number IACUC 16-BP-003. This study adhered to the ARRIVE Guidelines.

For each surgery, a standardized anesthesia protocol was applied. Inducing general anesthesia propofol was administered intravenously, and during surgery, 2–5% isoflurane was given via intubation tube. Before surgery, lidocaine/epinephrine was locally administered. After surgery, all animals received antibiotics (Enrofloxacin) for 7 days to prevent infections. The animals were monitored routinely after surgery, and analgesics were given if necessary.

### Surgical phases

**Fig. 1:**
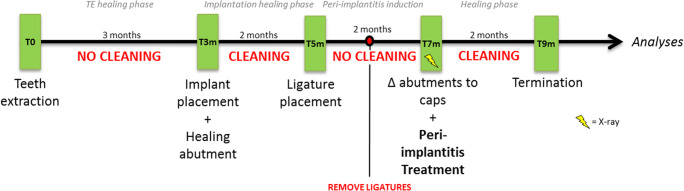
Flowchart of the experimental conditions and procedures

The different surgical interventions performed in each animal are described below, and Fig. 1 depicts the timeline of the experimental conditions and procedures.

#### Protocol of anesthesia

On the day of surgery, the animals were premedicated by intramuscular injection of atropine (0.05 mg/kg) followed by xylazine (2.7 mg/kg), and tiletamine-zolazepam (7.0 mg/kg intramuscular, Putney, Inc. Portland, ME), followed by mask inhalation of 2 to 5% isoflurane mixed with oxygen. Each animal was then transferred to the surgical suite, intubated with an endotracheal tube, and general anesthesia continued with 2 to 5% isoflurane mixed with oxygen. Directly before the surgical intervention, local injections of 1.0 mL/hemimandible of 2% lidocaine with epinephrine 1:50,000 (20 mg/mL + 12.5 μg/mL) into the buccal and lingual gingiva are performed to ensure total pain management.

#### Tooth extraction

In this first surgical step, P2–M1 were removed after reflection of full-thickness flaps and tooth separation. Primary wound closure was attained by means of mattress sutures, and the sites were allowed to heal for 3 months.

#### Implant placement

In a second surgical intervention, bilateral vestibular incisions were made, and full-thickness flaps were elevated to expose the edentulous sites for implant placement in the mandible. Two surgical implant beds were prepared bilaterally, at a distance of 8 mm apart, using a low-trauma surgical technique under copious irrigation with sterile 0.9% physiological saline. Two Bone Level Tapered SLActive titanium implants (BLT, SLActive) were placed in each side of the mandible (NC, Φ3.3 mm, length 8 mm, Institut Straumann AG, Basel, Switzerland) (*n* = 4 implants per dog) according to a one-stage procedure and covered with healing abutments (Ø 3.6 × H 2 mm Straumann, Basel, Switzerland, height: 5 mm). The implants were inserted in a way so that the borderline between the bony and transmucosal parts (BTB) of the implant coincides with the bone crest. Following irrigation, mucoperiosteal flaps were repositioned and primary wound closure achieved with interrupted resorbable sutures. Following implant placement, a plaque control regime was initiated. Tooth and implant cleaning were performed by the use of a toothbrush with chlorhexidine gel 4 times a week for 2 months.

#### Periimplantitis induction—ligature placement

In a third stage, 2 months after implant insertion, cotton ligatures were placed in a submucosal position around each implant by forcing them into an apical position. The ligatures were removed after another 2 months. Periimplantitis induction resulted in buccal bone defects at all implant sites.

#### Periimplantitis treatment and terminal procedure

The treatments were allocated to the implants randomly such that each treatment group was spread evenly across the different anatomical implant site positions.

**Fig. 2:**
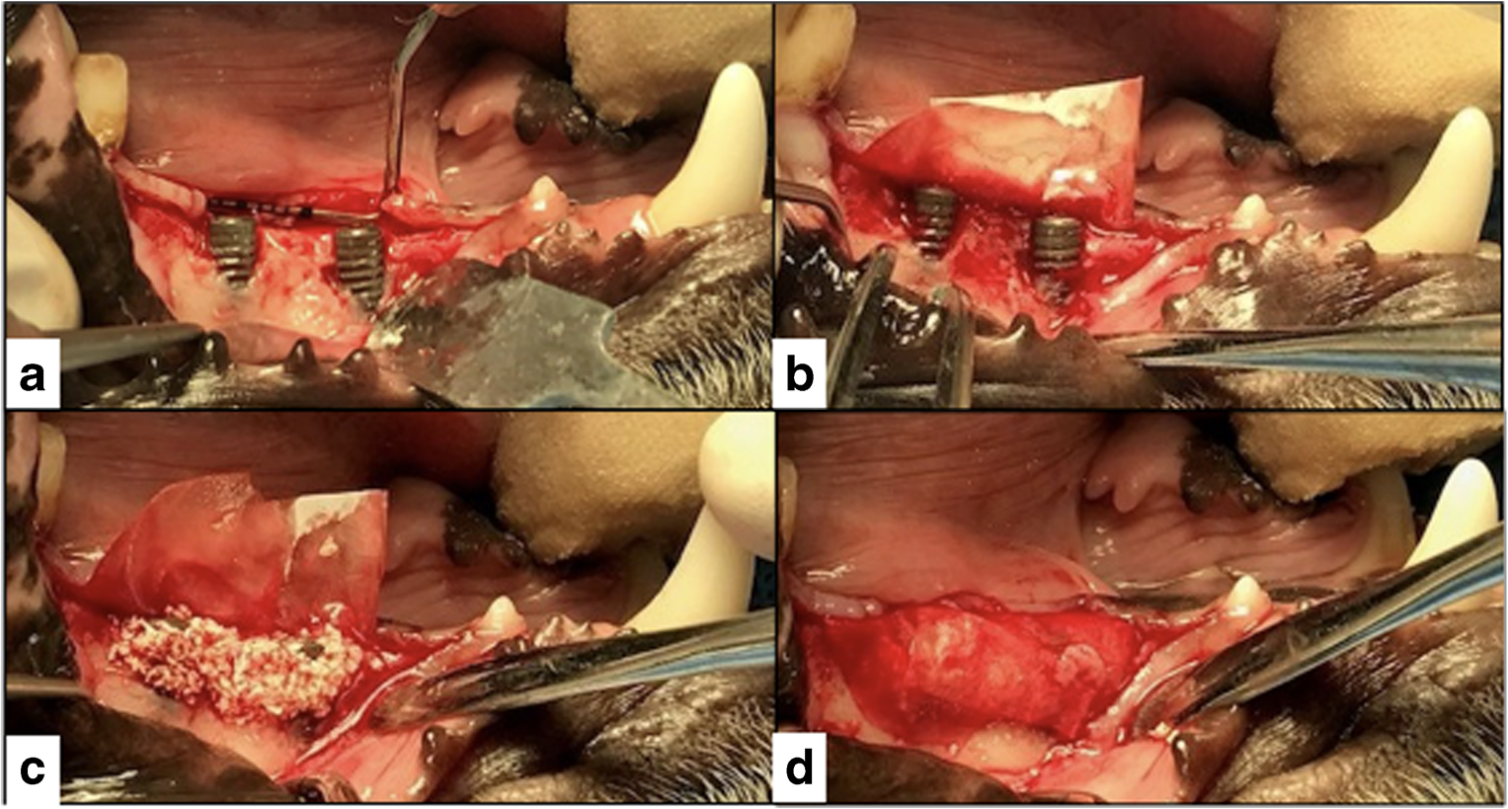
Representative clinical images of the performed treatments at the recession-type defects buccally after flap elevation and cleaning (**a**). Afterwards, the membrane was placed and secured at the lingual aspect (**b**) and the implant surfaces and defects were buccally filled with a xenograft (**c**). The membrane was adapted to cover the augmented sites (**d**), the periosteum was released and the site was closed for primary intention healing

During the fourth and final surgery, bilateral intrasulcular and vertical releasing incisions were made and full-thickness mucoperiosteal flaps were reflected to expose the respective periimplant bone defects. Intraoperative pocket probing depth measurements were conducted. All measurements have been carried out by the senior author (PRS), an experienced periodontist, with UNC15 periodontal probe (Hu-Friedy) probes in 0.5 mm increments; therefore, no intraexaminer calibration had to be performed. Following replacement of the healing abutments by cover screws (Institute Straumann AG), the granulation tissue was removed from the defects, and the respective implant surfaces were instrumented as follows: (1) mechanical debridement + GBR (DG) (control group), (2) air polishing using an experimental sterile powder (EMS, Nyon, Switzerland) + GBR (AG), (3) mechanical debridement + air polishing (DA), and (4) mechanical debridement + air polishing + GBR (DAG). The air polishing prototype device included a sterile single-use water and powderline, the first of this kind, and using a standard EMS AIRFLOW handpiece. Treatment was carried out as per recommendation usually done for subgingival treatment. After copious rinsing with saline solution of all the defect sites, in groups 1, 2, and 4, bovine bone mineral (BBM - Cerabone®, Botiss, Straumann AG, Basel, Switzerland) was filled into the remaining defect volume and covered by a barrier membrane (Jason®, Botiss, Straumann AG, Basel, Switzerland). The mucoperiosteal flaps were extended coronally to facilitate a submerged healing procedure. Primary wound closure was achieved with consecutive resorbable sutures. No randomization was performed (Fig. 2).

During the final healing phase, the animals received prophylactic antibiotic treatment for the first 3 days of the healing phase to avoid any surgical-related complications. Postoperative care consisted of chlorhexidine administration to the surgery site for 2 weeks postoperatively and then tooth brushing (2 times a week) for the remaining healing period. The total healing period was 2 months.

All animals were sacrificed 8 weeks after surgery 4. The termination was performed by inducing cardiac arrest with an intracardiac injection of a 20% solution of pentobarbital.

The block resection of the implant sites (mandible) was performed using an oscillating autopsy saw such that the soft tissue remained intact.

After the hemimandibles were isolated, they were fixed by immersion in formalin (formaldehyde 4% solution) for at least 2 weeks prior to sending for histological processing.

### Histology and histomorphometry

Histological processing and histopathologic evaluation was carried out by experienced pathologists (AnaPath Institute, Switzerland).

Bone samples (containing the implants) were immersed in formalin buffer solution, dehydrated using ascending grades of alcohol and xylene, infiltrated and embedded in metylmethacrylate for nondecalcified sectioning. Each site was cut in bucco-lingual direction. One section of 500 μm was obtained, grinded to a final thickness of 30–50 μm and stained with paragon (toluidine blue and basic fuchsin) for microscopic evaluation.

**Fig. 3:**
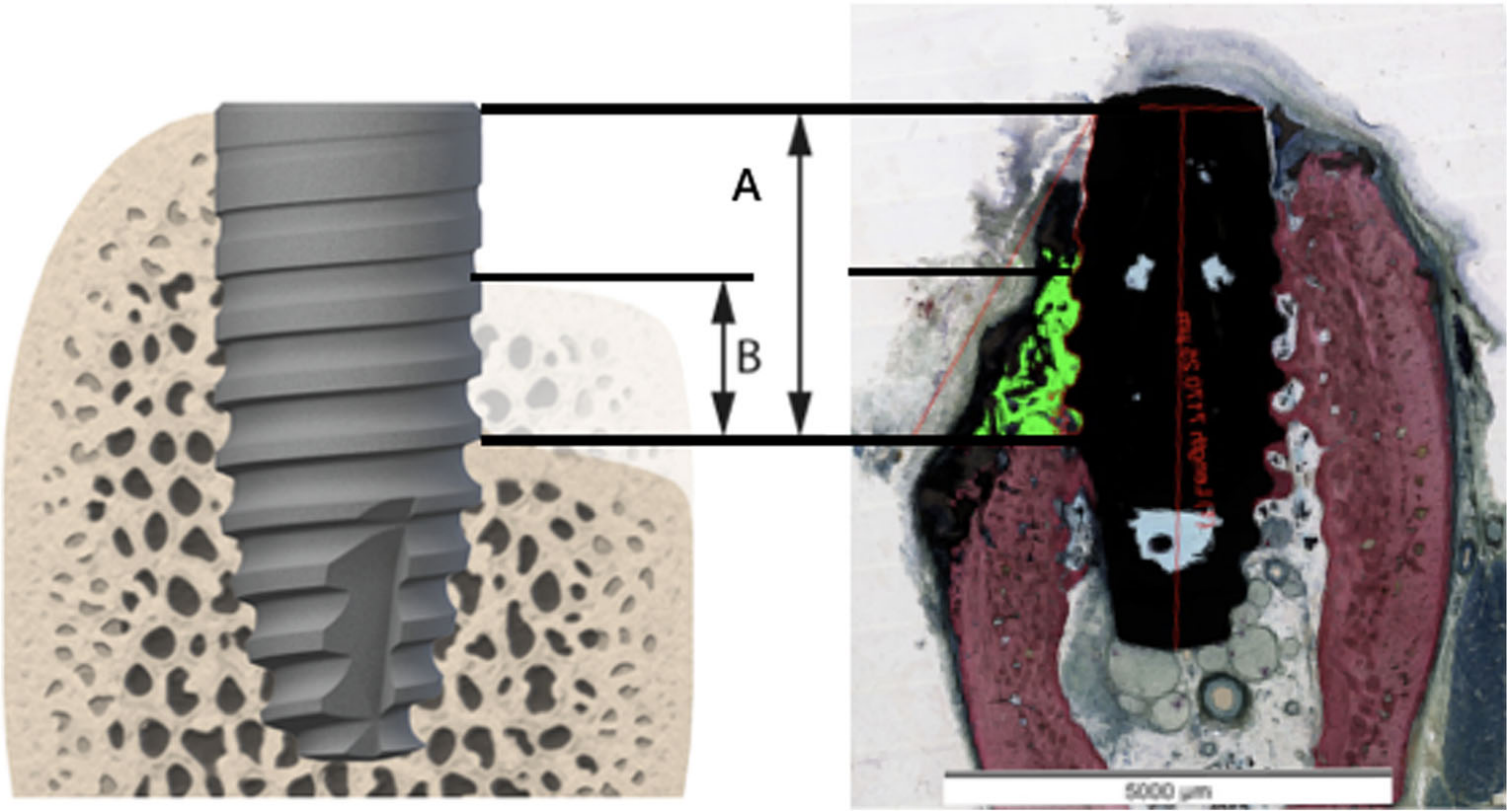
Histologic example and schematic illustration of the measurements. The green area elucidates the new bone. A Buccal defect depth before treatment. B Height of the newly formed bone after therapy (BIC)

Histomorphometrical measurements were done on central buccal sections within the region of interest (ROI) (Fig. 3). The depth of the buccal bone wall defects was determined by probing measurements taken intrasurgically at the time of treatment. After surgery defects were left to heal for a period of 8 weeks. To determine if the cleaning procedure was effective, a standardized ROI was defined on the histological slides by:

- The diameter of the sectioned implant was measured at its widest point (each implant was not necessarily sectioned perfectly down the middle. It is important to determine how much implant is represented in the histological image to ensure consistency in ROI area generations).
- Considering the ROI only extends apically to the probe depth measures of the bone defect, any bone within this ROI can be considered as new bone growth (green area) after the cleaning process (Fig. 3).
- All measurements regarding bone gain were measured linearly on base of the histomorphometric analysis (Fig. 3) with computer programs (Fiji and Image J, National Institutes of Health, Bethesda, MD, USA).

The following histological measurements were conducted at each implant (Fig. 3):

•Defect depth: distance from the implant shoulder to the most apical point of the periimplantitis defect before treatment (independently verified by two examiners).

• Newly formed bone: distance from the base of the defect to the most coronal bone-to-implant contact (BIC). Bone gain was measured in the absolute amount in millimeter (absolute bone gain).

The relative bone gain in relation to the initial defect depth has been calculated and evaluated accordingly.

Within the region of interest, defined by the implant position, the borders of the buccal defect and the course of the mandibular bone, area measurements of remaining bone substitute material (if applicable), newly formed bone, soft tissues, and tissue deficiency have been measured via computerized planimetry by a blinded examiner for all groups.

### Statistical analysis

The median values and IQR for the cumulative score are presented in a table (Tables 1, 3, and 4). The Wilcoxon’s signed rank test was used to evaluate differences between the treatments in the defect size recorded at the time of surgery and posttreatment. *P* values of less than 0.05 were considered statistically significant.

**Table 1:** Cumulative inflammation after different treatments according ISO 10993-6:2016(E) comparing the different treatments (for further details we refer to the respective “Materials and methods” section)

Treatment groups	Debridement + GBR (DG)		Debridement + air polishing + GBR (DAG)		Air polishing + GBR (AG)		Debridement + air polishing (DA)	
	Buccal	Lingual	Buccal	Lingual	Buccal	Lingual	Buccal	Lingual
Cumulative score	60	33	73.7	36.5	93.5	51.5	46	42
Group total	7	7	9	9	10	10	8	8
Average score	8.6	4.7	8.2	4.1	9.4	5.2	5.8	5.3

## Results

All dogs showed uneventful healing and survived all phases. Finally, a total of 33 out of 48 implants (7 DG, 9 DAG, 9 AG, and 8 DA) could be included in the final analysis. Most implants were lost during periimplantitis induction, i.e., before periimplantitis treatment was started. After therapy, only two implants were actually lost (both in the DG group). Three implants (all in one dog; no. 10) were excluded due to insufficient bone integration at the buccal aspect, i.e., implants were completely outside the bony envelope (1 DAG, 1 DG, 1 DA).

### Histopathology

In general, no inflammation was noted at periimplant tissues representing successfully (re)osseointegrated aspects. Bone growth was nicely joining the implant distance within the mandibular bone tissue. Fibrosis and inflammatory infiltrate were mainly noted at those sites, which achieved no bone gain; nevertheless, biofilm was not visually detectable (histology not shown).

At the lingual aspect, in general low irritation scores were measured ranging from 5.8 (DA - debridement and air polishing) to 4.1 (DAG - debridement and air polishing and GBR). At the buccal side, score ranged from 5.8 (DA - debridement and air polishing) to 9.4 (AG - air polishing and GBR) with no significant differences between all groups. However, with regard an additional “irritative potential” according to ISO 10993-6:2016 (E), mean differences between test items above 2.9 can be considered indicating an inflammatory implication [13]. Therefore, comparison at the lingual aspects showed no notable irritation potential or difference between groups, whereas a difference of 3.6 was considered a slight irritative potential as compared with the group with the lowest score in the control (DA - debridement and air polishing). The rather low score in the latter group was mainly due to low fibrosis, neovascularization, and mononuclear cells. In all other groups, the higher inflammation scores were influenced mainly by two factors, i.e., the presence of bone substitute particles and the implant setting in an oblique direction. On a cellular base, inflammation was characterized through the presence of polymorphonuclear cells, lymphocytes, macrophages, and plasma cells.

**Fig. 4:**
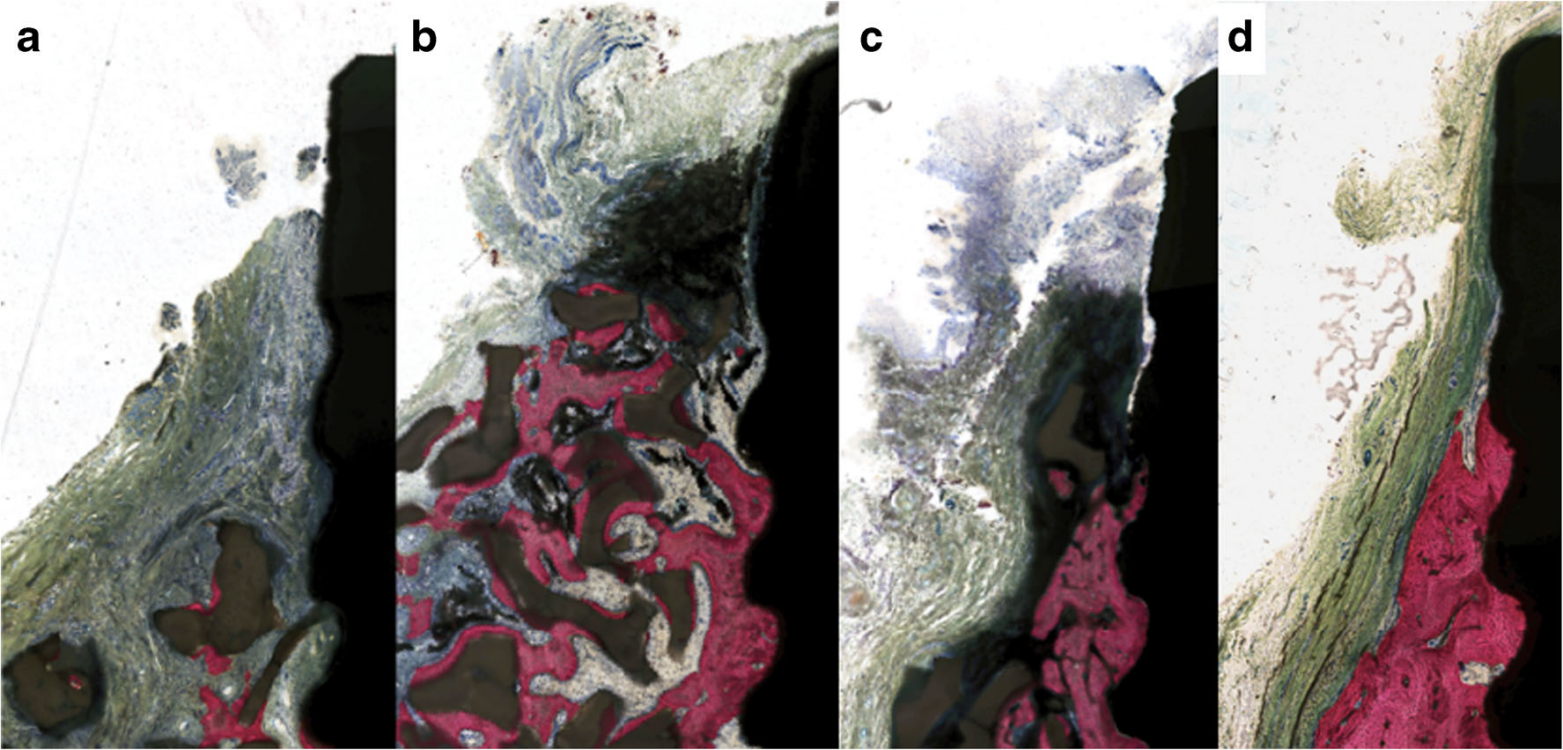
Representative histologies of the buccal aspect for each treatment group: **a** debridement and GBR, **b** air polishing and GBR, **c** debridement air polishing and GBR, **d** debridement and air polishing

GBR procedures apparently resulted in significantly higher (*p* < 0.05) inflammatory scores at the buccal sites as compared with ungrafted ones (5.8 vs. 8.2–9.4). Histologically, the bone substitute material was not fully integrated into the bony tissue, and mainly in the gingival tissues, this caused a more accentuated inflammatory infiltrate accompanied by surrounding fibrotic changes. Figure 4 shows representative probes for all four treatment groups. Table 2 is elucidating cumulative score of the inflammatory cells, which corroborates a slight increase of lymphocytes in grafted sites.

**Table 2:** Cumulative score of inflammatory cells in each group at the buccal (augmented) sites according to ISO 10993-6:2016(E)

Cell type		Treatment			
		DG	DAG	AG	DA
1	Polymorphonuclear cells	0.1	0.1	0.3	0
2	Lymphocytes	1	1.1	1.3	0.4
3	Plasma cells	0	0	0	0
4	Macrophages	0.1	0.1	0.1	0

### Bone gain after treatment

No significant differences could be observed between treatment groups with regard to the initially created periimplantitis defect characteristics (Table 3): Buccal bone defect depths ranged between 3.5 mm (DA - debridement and air polishing) and 4.5 mm (DAG - debridement and air polishing and GBR and AG – air polishing and GBR).

**Table 3:** Measurements of the defect depths (A) and the respective absolute bone (B) in mm as well as the relative bone gain (B/A) in percent according to figure 3 (median and IQR (in brackets) are presented for each group)

Group	DG (*n* = 7)	DAG (*n* = 9)	AG (*n* = 9)	DA (*n* = 8)	*p* Value (stat. sig.)
A					
Defect depth (mm)	4.0 (1.8)	4.5 (2.5)	4.5 (1.0)	3.5 (2.3)	n.s.
B					
Bone gain (mm)	1.21 (0.94)	1.98 (0.90)	2.65 (2.45)	1.73 (1.05)	n.s.
Relative bone gain (%)	39% (34)	53% (17)	59% (44)	53% (29)	n.s.

Regarding new BIC, no statistical difference was detected between the different treatment groups. No implant showed complete healing of the defect. Absolute bone gain ranged between a median of 1.2 mm (debridement and GBR) and 2.7 mm (air polishing and GBR). The relative bone gains ranged from a median of 39% (DG - debridement and GBR) to 59% (AG - air polishing and GBR), respectively (Table 3).

### Area measurements

Area measurements performed on a preset area of interest including remaining substitute material, new bone tissue, soft tissue, and tissue deficiency resulted in a mean new bone regeneration of 19–47.8% between the different groups. In the three groups using bone substitute materials (DG, DAG, DG), the remaining substitute materials of 11 to 21.5% could be detected within the region of interest. Soft tissue ranged from 52.9 to 61.9% with the highest values in the DG group. Some implants showed soft tissue deficiencies resulting in recessions ranging in a mean of 2.3 to 8.2%. Statistical no significant differences could be found (Table 4).

**Table 4:** Mean (SD) + median (IQR)—values in % for the prevalence of histologic measurements in the predefined region of interest (rounded to the first decimal digit after the comma). Significancy assessed through Wilcoxon’s signed rank test

Group	DG (*n* = 7)	DAG (*n* = 9)	AG (*n* = 9)	DA (*n* = 8)	*p* Value (stat. sig.)
Substitute material (%)	14.2 (6.6) 14.8 (5.1)	11.0 (8.0) 9.6 (5.7)	21.5 (12.3) 24.6 (15.2)	NA	n.s.
Bone tissue (%)	19.0 (10.9) 22.3 (17.3)	27.9 (14.4) 27.2 (18.7)	19.8 (13.2) 21.4 (23.4)	47.8 (22.6) 50.2 (27.3)	n.s.
Soft tissue (%)	61.9 (11.8) 57.0 (16.3)	52.9 (19.5) 56.9 (23.7)	55.7 (12.8) 53.7 (13.6)	53.6 (18.6) 48.5 (16.3)	n.s.
Tissue deficiency (%)	4.8 (10.4) 0.0 (3.7)	8.2 (12.1) 0.0 (14.1)	3.0 (5.1) 0.0 (4.0)	2.3 (3.5) 0.0 (4.6)	n.s.

## Discussion

The aim of the present study was to compare exemplary intrasurgical antiinfective protocols alone or in combination with GBR when treating periimplantitis defects in a dog model. Disinfection of the surgically exposed implant surfaces was achieved using air polishing and/or debridement with a carbon curette. GBR in this study consisted of the application BBM in combination with a resorbable collagen membrane.

All treatment approaches resulted in a partial regeneration of the defects. But no treatment group showed a significantly better outcome. However, pretreatment with air polishing showed a tendency towards less inflammation, and—noteworthy—inflammation assessment showed rather an irritative potential after GBR in the evaluated early healing phase.

So far, several antiinfective protocols have been proposed to primarily clean and disinfect the exposed dental implant in the management of periimplantitis [9]. The usage of air polishing using glycine powder against biofilms at implants was already described in vitro and represents one of the most promising methods as compared to curette or sonic-scaler debridement [14, 15]. A bacterial reduction of 99.9% on implant surfaces using different systems of air polishing has been shown [16]. Further air polishing was already used and described as a decontamination protocol in a periimplantitis dog study in 1997 [17]. The surgical protocol in the current study includes the use of an experimental sterile bicarbonate-based powder. To date, the use of sterile powders has never been described in a preclinical study marking a novelty in biofilm disruption and periimplantitis therapy.

Glycine powder used in the cited studies is a PERIO powder with a mean size of about 20–25 microns. Generally, sodium bicarbonate is of bigger size (40–65 microns) for supragingival cleaning, but in this study, an experimental sodium bicarbonate powder with a mean particle size of 12–18 microns was used. Two reasons led to this decision: first reducing the size reduces the abrasivity of sodium bicarbonate powder to reach level similar to glycine PERIO powder. Secondly, the possibility to sterilize this sodium bicarbonate powder arose.

Furthermore, when it comes to open-flap surgery for the treatment of periimplantitis, sodium bicarbonate air polishing has been found to be an efficient tool to remove biofilms and has been verdicted as safe when used properly [18]. This could relate to the fact that periimplantitis surgery, as described in this paper, does more correspond to a supragingival use as a full-thickness flap is elevated. Nevertheless, here, the small-sized sodium bicarbonate powder could combine the advantage of the efficiency and the low abrasivity. Additionally, the usage of this small-sized sodium bicarbonate powder relays on the air polishing studies using sodium bicarbonate to decontaminate implant surfaces. In vitro it was shown that thorough removal of biofilms on implant surfaces can be achieved [18]. In 2011, Schwarz et al. [19] have shown that sodium bicarbonate based air polishing does not lead to significant alterations of the implant surface or coating. In in vitro studies, the biocompatibility of the titanium surface after air polishing with sodium bicarbonate has been described as very high [18].

The ligature-induced periimplantitis model in this study has been extensively used in periimplant disease studies observing the pathophysiology and testing the efficacy of different therapeutic protocols [17, 20–22]. After 2 months of ligature placement, not rather class-I-e-defects, as mostly described [23], but rather buccal dehiscence class-I-a-defects, were observed [23]. The authors attribute this finding on the one hand to the size of the included beagle dogs and the resulting small anatomy including ridge width of the mandibles compared to the implant diameter and on the other hand—as already been described—that the evaluated bone level implants as such show only minor defect progression and defect formation in animal periimplantitis models [24]. This fact might have also led to the relatively high number of lost implants (10 implants) during healing phase. Similar defect morphologies were also observed in a recent periimplantitis dog study [25].

The resulting dehiscence defects were treated with four different approaches. The different approaches led to improved clinical outcomes and bone gain. But none of the implants showed a complete resolution of the defects. Relative resolution of the defects resulted in 39–59%. Despite a large median difference of 20%, no statistical significance between the groups was shown. This relates mostly to the high statistical variance within the groups. No power analysis could have been carried out before due to a lack of comparable date. However, a quite large sample of 12 dogs including 33 implants appeared to be an adequate sample size as compared with other periimplantitis dog studies. Not more than tendencies to an improved bone gain are pointing towards treatment methods with the use of air polishing with obtained bone gain values of 53–59% as compared with procedures without. Surprisingly, the control group without any GBR-procedures (debridement and air polishing) showed similar results to the DAG group (debridement and air polishing and GBR) and even better results compared with DG (debridement and GBR) alone after 2 months of healing.

Two-dimensional area measurements of a predefined region of interest further mirrored results in terms of new bone formation. DA group without any GBR procedures resulted in a mean of 47.8% of newly formed bone, while GBR groups showed only 19–27.9% of real new bone. Adding the remaining bone substitute particles in the region (11–21–5%), all groups show a similar distribution of augmentated hard tissues and soft tissues. Some implants showed a partial tissue deficiency in the region of interest resulting in a clinical recession.

Findings without additional benefit of GBR procedures in periimplantitis therapy in dogs were also described in a similar study comparing two antimicrobial protocols with and without GBR [26]. Further, Nociti et al. [27, 28] in 2000 and 2001, made the same observations concluding that GBR in periimplantitis defects in dogs does not lead to significant advances in terms of new bone formation. These findings stand in contrast to a study by Hürzeler et al. [17] in 1997, where additional bone gain through GBR compared with no regeneration procedure was shown. It has always to be kept in mind that the present study is assessing buccal dehiscence defects while most other studies describe saucer-shaped defects [17, 27, 28], hindering a direct comparison.

As an important finding of this study, it was shown that the groups treated with a combination of bone regeneration procedures with bone substitute materials in the first weeks cause a higher degree of inflammation compared with no GBR procedures, leading to the conclusion that bone substitute particles lead to enhanced tissue inflammation in early wound healing. This process was considered a physiological reaction and to be subject of resolution during the following months. Inflammation scoring shows findings of doubtful clinical significance. However, it can be shown that the preclinical usage of the experimental sterile powder during open surgical treatments is not causing any inflammation and can be verdict as safe.

Three implants have been excluded of the analysis (dog no. 10), as all have been described bad positioned outside the bony envelope. The implantation caused at several implantation sites a massive destruction of the buccal bone tissue due to rather small alveolar bone width at the time of implantation. The implant at this site was mainly surrounded by previously existing connective tissues but not surrounded by bone tissue. Hence, the reaction was deemed to be the result of an inadequate technical issue related to the dog mandibular bone and the implant/drill diameter rather than a material-related finding. This led to an impossible situation for bony regeneration, favoring fibrosis and inflammation in the healing.

Weak points of the study are clearly the high statistical variance of the results despite the relatively high amount of test animals and the powerful within-subjects design, leading to statistical not significant and thus unfortunately not entirely conclusive result. Finally, the authors suggest further research with air polishing powders and GBR in human, as the use of air polishing can be considered safe and does not hinder grafting procedures.

## Conclusion

Within the limits of this animal study, it can be concluded that:

- All treatment approaches resulted in a certain degree of bony regeneration of the buccal dehiscence.
- Air polishing cleaning resulted in a tendency to a greater new bone healing as compared with no air polishing, though not statistically significant.
- Considering inflammatory infiltrate after cleaning, no significant differences were found between the groups.
- Preclinical use of sterile air polishing powder and a sterile delivery system in open surgical treatment around implants does not result in an immune reaction considered to be irritating and can thus be considered safe, as per ISO 10993-6:2016. Though augmentation procedures in previously infected and exposed sites may cause more inflammatory response than cleaning alone during early healing.
